# The road not taken: Could stress-specific mutations lead to different evolutionary paths?

**DOI:** 10.1371/journal.pbio.2002862

**Published:** 2017-06-08

**Authors:** Deepa Agashe

**Affiliations:** National Centre for Biological Sciences, Bangalore, India

## Abstract

Organisms often encounter stressful conditions, some of which damage their DNA. In response, some organisms show a high expression of error-prone DNA repair machinery, causing a temporary increase in the genome-wide mutation rate. Although we now have a detailed map of the molecular mechanisms underlying such stress-induced mutagenesis (SIM), it has been hotly debated whether SIM alters evolutionary dynamics. Key to this controversy is our poor understanding about which stresses increase mutagenesis and their long-term consequences for adaptation. In a new study with *Escherichia coli*, Maharjan and Ferenci show that while only some nutritional stresses (phosphorous and carbon limitation) increase total mutation rates, each stress generates a unique spectrum of mutations. Their results suggest the potential for specific stresses to shape evolutionary dynamics and highlight the necessity for explicit tests of the long-term evolutionary impacts of SIM.

The evolutionary trajectories of organisms are paved with mutations, which generate the raw material (genetic variation) essential for evolutionary change. In biology class, we learn that mutations are random: the probability that a mutation occurs is independent of its fitness effect (i.e., its impact on individual survival or reproduction). As Luria and Delbruck famously showed [1], bacterial mutations that confer resistance to a virus continually arise in a population before exposure to the virus; under subsequent viral infection, these mutations spread in the population. Thus, mutations do not occur in response to a specific stress once it is encountered, and the fact that we observe beneficial mutations after imposing selection is simply an outcome of natural selection. Clearly, genetic variation is critical for adaptation, so why don’t mutation rates continually increase? In fact, very few mutations are beneficial; most have no phenotypic effect or are deleterious (see [2] for a recent review). Thus, increasing the total mutation rate would introduce a “genetic load” in the population, whereby most individuals will acquire deleterious mutations. The problem is exacerbated in asexual populations by Muller’s ratchet (the irreversible accumulation of deleterious mutations): without sex, mutation-free regions of two genomes cannot recombine and generate mutation-free offspring. Indeed, in laboratory experiments, bacteria with high mutation rates (“mutators”) may have a growth advantage in the short term, but as they accumulate deleterious mutations, they are eventually outcompeted by nonmutators (e.g., [3]). Hence, selection should generally act against increasing mutation rates. Additionally, adaptation may not always be limited by the supply of mutations: standing genetic variation (i.e., already existing mutations, expected in large populations) may include beneficial genotypes that allow for rapid adaptation to environmental change [4].

However, a large body of work demonstrates stress-induced mutagenesis (SIM)—a transient increase in mutation rates under stresses such as antibiotic exposure or starvation—via specific pathways that are typically suppressed under rapid growth [5,6]. This has been best studied in bacteria, where stress-associated DNA damage triggers the SOS response, a large and complex network of genes and proteins that pauses cell division and repairs damaged DNA [7]. The response includes upregulation of the RpoS protein, a global regulator of gene expression. The regular high-fidelity, methyl-directed mismatch repair pathway (MMR) is suppressed, and error-prone DNA repair machinery (involving DNA polymerase IV and V) is upregulated, ultimately increasing the mutation rate (Fig 1A). Analysis of single genes suggest that SIM can also alter the mutation spectrum [8]—the relative frequency of different types of mutations (e.g., base pair substitutions or large insertion sequences)—thus, determining the available set of mutations upon which natural selection can act. *E*. *coli* strains lacking MMR genes also show distinct mutation spectra [9,10], indicating that the suppression of MMR induced by stress may have a similar effect. In fact, inducing high RpoS expression in nonstressed *E*. *coli* also significantly changed the mutational spectrum [11], suggesting that the perception of stress (despite growing in rich media) is sufficient to alter mutation spectra. SIM occurs in diverse organisms and contexts, including natural bacterial isolates [12] and cancer cells [13]; in many of these cases, the mechanisms underlying SIM are well understood. However, the potential implications and interpretations of SIM for evolutionary dynamics have been controversial. Does SIM represent an adaptive mechanism to increase sampling of beneficial mutations under stress, or is it a by-product of a tradeoff between efficient and accurate DNA repair as suggested by the crystal structure of the error-prone polymerase [14]? At the heart of this debate are various open questions (Fig 1B) whose answers are critical to determine the evolutionary implications of SIM. For instance, which stresses induce mutagenesis, and are the mutational consequences consistent across stresses?

**Fig 1:**
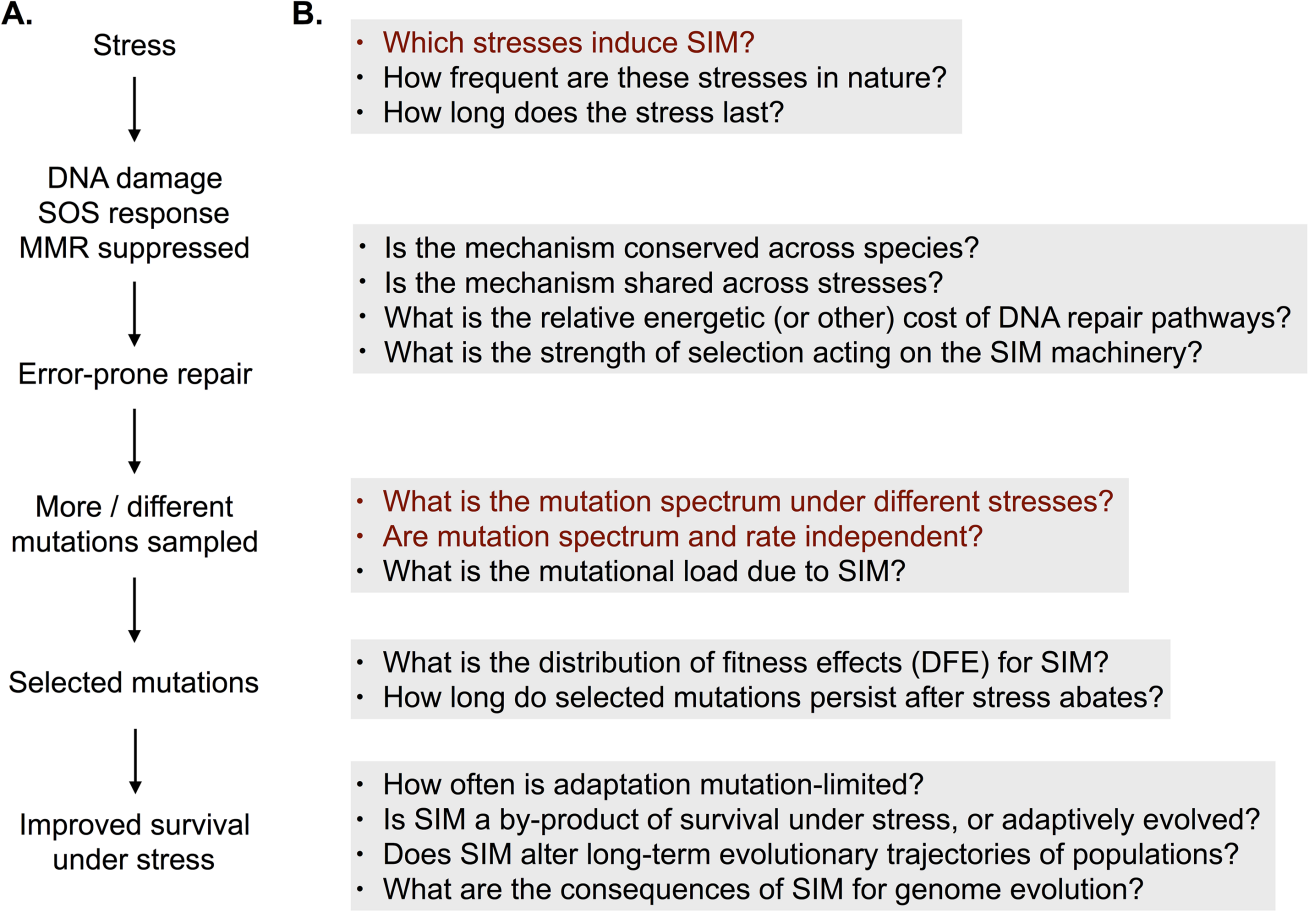
(A) Summary of key aspects of stress-induced mutagenesis (SIM), and (B) open questions relevant for each step. Questions addressed by Maharjan and Ferenci [15] are highlighted in red. MMR, mismatch repair.

In this issue, Maharjan and Ferenci present a study that addresses these key questions by determining the type and location of mutations under various (largely nutritional) stresses: phosphorous (P), carbon (C), iron (Fe), oxygen (O), and nitrogen (N) [15]. Measuring SIM-associated mutation rates and spectra is challenging because it is difficult to maintain a constant environment and minimize the impact of selection during the measurement. To overcome these problems, Maharjan and Ferenci allowed cycloserine-sensitive (Cyc^S^; sensitive to the antibiotic cycloserine) *E*. *coli* to grow in a chemostat. The chemostat ensured a constant flow rate of growth media, maintaining the concentration of each limiting nutrient and reducing growth rate by ~7-fold compared to nutrient excess. To minimize the effects of selection, they allowed populations to experience the nutrient stress for a relatively short period of 72 hours. They then assayed populations for cycloserine resistance (Cyc^R^) and sequenced the responsible gene (*cycA*) to determine mutation rates and spectra. Importantly, the cycloserine resistance/sensitivity phenotype has no fitness impacts under the experimental conditions, and the measured mutation rates and spectra were thus independent of selection.

Maharjan and Ferenci found that stress-induced mutation spectra were stress-specific, and that only some stresses were associated with increased mutation rates. P and C starvation induced a 4- to 9-fold increase in the total mutation rate per locus per generation, whereas N, O, and Fe starvation had no impact on the mutation rate. Interestingly, regardless of the total mutation rates, the mutation spectra differed substantially across all stresses. For instance, insertion sequence transpositions were higher in Fe and O starvation compared to C and N, and lowest under P limitation. P limitation also induced a large overproduction of base pair substitutions, primarily due to GC→AT transitions and GC→TA transversions. Thus, each stress produced a unique set of mutations that were then available for subsequent evolution. Importantly, the mutational spectra inferred from *cycA* correctly predicted the frequency of phenotypic changes expected from mutations in three other genes (*lacZ*, *bgl*, and *araD*). For instance, since frameshifts in *cycA* were more likely under P limitation, we would expect that phenotypic changes resulting from frameshifts in other genes would also arise more often under P limitation. Supporting this expectation, the araD+ phenotype (arising due to a +1 frameshift in *araD*) was observed more frequently under P limitation, despite no fitness effect of the mutation under P starvation. Whether these altered mutational probabilities are relevant for subsequent adaptation to P limitation remains to be tested.

Maharjan and Ferenci’s study poses new questions whose answers should lead to further insights about the evolutionary consequences and interpretations of SIM. For instance, what is the mechanistic basis of the stress-specific mutation spectra? Previous work has largely focused on antibiotic stress and total nutrient starvation, so relatively little is understood about the stress response pathways under the specific nutrient depletion tested in this study. The simplest explanation may be differential upregulation of various error-prone DNA repair enzymes under various stresses. Maharjan and Ferenci found that compared to an unstressed control, RpoS was upregulated in all stress treatments, as was the error-prone polymerase Pol IV. Despite this, there was no evidence for increased mutation rates in three stresses (Fe, N, and O limitation). Clearly, other pathways are also important for SIM under these stresses, and the detailed molecular mechanism responsible for stress-specific mutation spectra needs to be tested.

A second question concerns the evolutionary impact of the altered mutation spectra. The evolutionary fate of mutations depends on population size as well as the fitness impact of mutations, which is summarized by the frequency distribution of fitness effects (DFE). Typically, the DFE is heavily biased towards neutral and deleterious effects [2,16]. If altered mutation spectra skew this distribution towards beneficial mutations, the mutational load associated with increased mutation rates may be lower. In MMR-deficient *E*. *coli* strains, distinct mutation spectra do result in altered DFEs and short-term competitive ability [10]; more generally, the shape of the DFE and the expected fraction of beneficial mutations varies across environments and across species [17,18]. Hence, it is important to know whether stress-specific mutation spectra also result in stress-specific changes to the DFE, and whether this improves long-term adaptation to the particular stress. Previously, Torres-Barceló and colleagues allowed a SIM-deficient mutant lacking an SOS response to compete with wild-type (WT) *Pseudomonas aeruginosa* under antibiotic (ciprofloxacin [Cip]) stress [19]. They found that the WT outcompeted the mutant in the short-term, and continued to show slightly faster adaptation to Cip. However, within 200 generations, both WT and mutant converged on similar fitness on Cip, indicating that the advantage of a functional SOS response (and associated high mutation rate) is relatively short-lived. It remains unclear whether these results are generally true under different stresses and across genetic backgrounds. For instance, do mutants isolated during a short-term carbon stress underperform under phosphorous stress, and vice versa? Finally, it is worth noting that we have a very poor understanding about the relative frequency, duration, and potential co-occurrence of various stresses. If nutritional, antibiotic, or biotic stresses are rare, transient, or occur independent of each other, selection favoring SIM is expected to be relatively weak and it is more likely to evolve under genetic drift [20]. Maharjan and Ferenci’s work suggests that analyzing SIM under ecologically relevant stresses is critical to clarify the broader evolutionary role of SIM.

Beyond SIM, Maharjan and Ferenci’s work also has implications for other aspects of molecular evolution. Their results suggest that bacteria exposed to a repeated, specific nutritional stress may accumulate distinct mutations over long periods, potentially affecting genome architecture over time. Such genome-level impacts are important to better understand the evolution of bacterial genomes. For instance, the relative role of genetic drift versus selection in driving evolutionary change in genomic nucleotide content (which varies from ~13% to ~75% GC across bacteria) has been debated for decades [21]. Previous work suggests that high GC content may be selectively favored under N limitation [22]. Maharjan and Ferenci’s results suggest that N starvation may also directly alter GC content by changing mutation spectra even before selection acts. Thus, organisms’ niches may directly influence mutational availability, shaping the substrate available for natural selection and determining genome evolution. The critical next step is to experimentally test these potential, wide-ranging evolutionary implications of stress-specific mutagenesis.

## Abbreviations

C: carbon
Cip: ciprofloxacin
Cyc^R^: cycloserine resistant
Cyc^S^: cycloserine sensitive
DFE: distribution of fitness effects
Fe: iron
MMR: mismatch repair pathway
N: nitrogen
O: oxygen
P: phosphorous
SIM: stress-induced mutagenesis
WT: wild-type
